# A rare laryngeal tumor in a patient with thyroid papillary cancer: granular cell tumor^☆^

**DOI:** 10.1016/j.bjorl.2015.12.007

**Published:** 2016-03-29

**Authors:** Sheng-Yao Cheng, Li-Hsiang Cheng, Yi-Shu Liao, Wen-Sen Lai

**Affiliations:** a National Defense Medical Center, Tri-Service General Hospital, Department of Otolaryngology–Head and Neck Surgery, Taipei, Taiwan; b National Defense Medical Center, Tri-Service General Hospital, Department of Pathology, Taipei, Taiwan; c Taichung Armed Forces General Hospital, Department of Otolaryngology-Head and Neck Surgery, Taichung, Taiwan

## Introduction

Granular cell tumor (GCT), Abrikossoff's tumor, is a rare tumor, especially that occurring in the larynx. It may share similar appearance and symptoms with laryngeal granuloma, polyp, or nodule although they have different etiology and features (Table 1). The diagnosis must be made by histological examination. In the past decades, the origin of GCT remained controversial but current mainstream opinion regarded it as of neuroectodermal origin according to immunohistochemical staining.^1^ Here we presented a patient with a history of papillary thyroid carcinoma under regular follow-up who was diagnosed recently with a GCT of the larynx. The related literatures were also reviewed.

**Table 1:** Vocal fold lesions.

Vocal fold lesions	Features	Common causes	Treatment
Nodule	Usually bilateral	Chronic voice abuse	Speech therapy
	Smaller		Excision

Polyp	Often unilateral	Acute voice abuse	Behavior modification (quit smoking, speech therapy)
	Obvious surface	Chronic irrigation (For example, smoking, gastroesophageal reflux)	
	Blood vessel		Excision

Granuloma	Often bilateral	Repeated trauma (For example, intratracheal intubation)	Excision
	Larger		

Granular cell tumor	Single	Unknown	Excision
	Unilateral		
	Most common at posterior glottis		

## Case report

**Figure 1:**
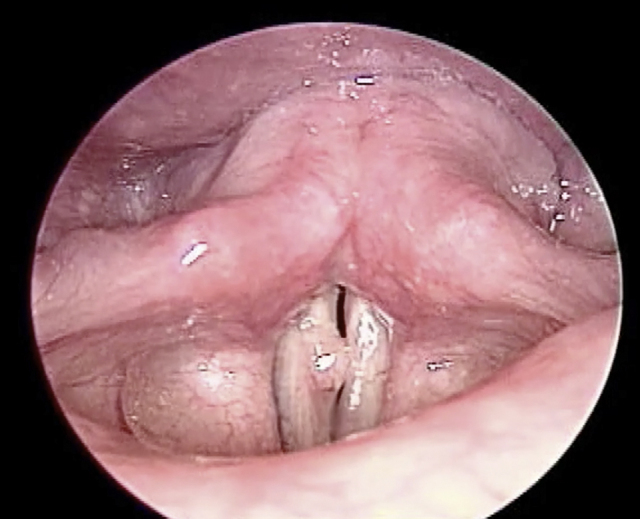
Preoperative indirect laryngoscope finding: a white tumor lesion was at right vocal fold with mucosal cover and unclear border which led to failure of glottis closure during phonation.

**Figure 2:**
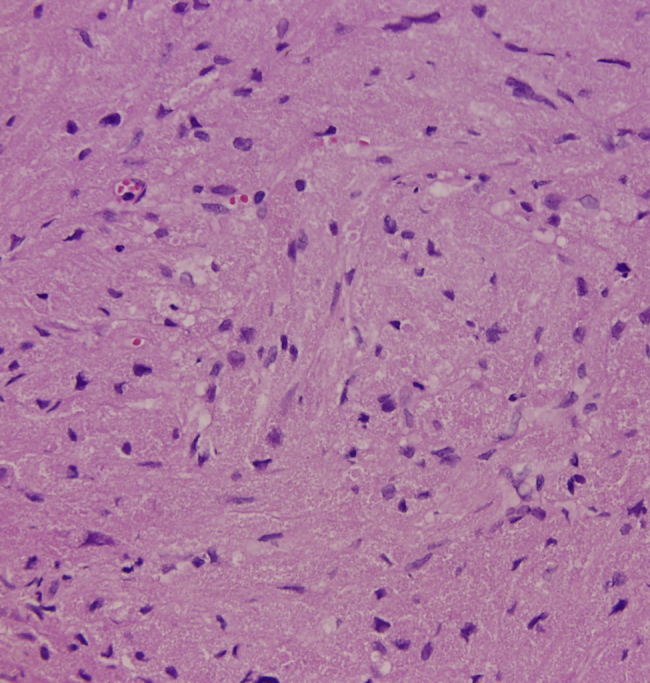
The tumor comprised round or polygonal cells with abundant eosinophilic granular cytoplasm and small uniform nuclei arranged in nest and sheet patterns (H&E, 400×).

A 35-year-old female presented with a 10 month history of progressive painless hoarseness and easy voice fatigue. Three years ago, she was diagnosed with papillary thyroid cancer and received total thyroidectomy and Iodine-131 therapy. Since then, she has been under daily levothyroxine supplement (0.1 mg per day) and regular follow-up. She has been smoking 20 cigarettes per day over 10 years but without voice abuse history. There was no palpable neck mass on physical examination. Due to the persistent symptom, she came to our clinic where indirect laryngoscope showed a white tumor lesion at right posterior third vocal fold with mucosal cover and unclear border which led to failure of glottis closure during phonation (Fig. 1). Because of her history and the appearance of tumor, primary laryngeal malignancy or recurrent papillary thyroid carcinoma metastasis was suspected. The tumor was completely excised through microlaryngeal surgery with cold knife under general anesthesia. The lesion measured about 0.5 cm × 0.2 cm in size and was totally embedded for sections. Histopathologic examination revealed one nodular lesion with pseudoepitheliomatous hyperplasia. Under hematoxylin-eosin (H&E) staining, polygonal cells with abundant eosinophilic granular cytoplasm and small uniform nuclei arranged in nests was impressed (Fig. 2). There was no evidence of malignancy due to the absence of pleomorphism with small nuclei.

**Figure 3:**
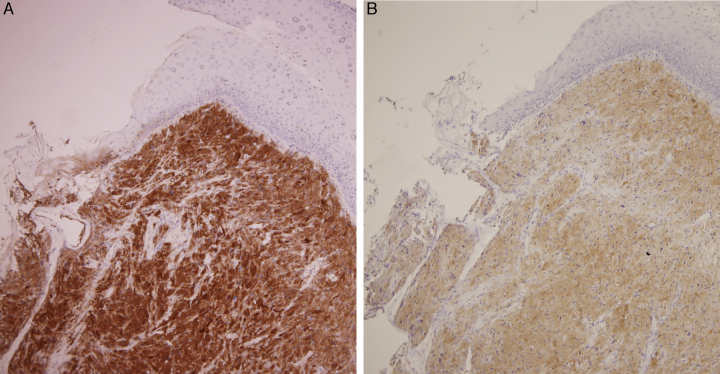
(A) Positive S-100 immunostain. (B) Positive neuron-specific enolase immunohistochemical staining denoted its neuroectodermal origin.

Immunohistochemical staining showed positive for S-100 protein (Fig. 3A), vimentin and neuron-specific enolase (Fig. 3B). The tumor was uniformly negative for thyroglobulin, TTF1, and HBME-1. According to the above pathologic results, the final diagnosis was laryngeal granular cell tumor. After 6 months of follow-up, she was satisfied with her voice outcome and no local tumor recurrence was noted.

## Discussion

Most cases of papillary thyroid cancer had laryngeal involvement through direct invasion and were regarded as advanced stage. Although papillary thyroid carcinoma tends to spread via the lymphatic system, manifesting as laryngeal metastatic nodules, hematogenous spread had also been reported before.^2^ The patient had a thyroid cancer history and was under regular follow-up after total thyroidectomy. Thus, metastatic malignancy should be considered for differential diagnosis of laryngeal tumors.

GCTs are uncommon neoplasms and can be found in any organ of the whole body. About half of the cases are found in the head and neck, with the tongue being the site most affected in this region. The larynx being affected by GCT is as rare as 3–10% of all cases.^3^ Different from other common laryngeal lesions such as polyp or nodule, GCT showed slight female predominance, and no causal relationship between GCT and voice abuse had been established.^4^ The symptoms of GCTs vary with tumor size and localization. Weakening hoarseness is the most common symptom. However, lump sensation in the throat, dry cough, hemoptysis, and odynophagia can also be present.^5^ Grossly, laryngeal GCTs are characterized as being firm, round, mucosa-covered masses located most frequently in the posterior two-thirds of the vocal folds although other sites such as arytenoid, posterior cricoid region, supra- or subglottic areas have also been described.^6^

They often resemble vocal fold granulomas, polyps, and even malignant lesions. Consequently, the definite diagnosis was made by histopathological examination which demonstrated polygonal cells with thickening of the cell membrane and abundant eosinophilic intracytoplasmic granules. The origin of GCT remained controversial. It was first described by Abrikossof, in 1926, who named it as myoblastoma that was considered from skeletal muscle cells base on their cytologic picture.^7^ However, recent immunochemistry studies have provided better evidences of the origin of this tumor which show positive for S-100 protein, neuron-specific enolase, and CD68 but negative for muscular markers such as myoglobin, keratin, and desmin. These staining characteristics suggested that they originate from the neuroectodermal tissue or Schwann cells rather than muscle cells.^4^ In recent immunohistochemical study, GCTs have a component of endomesenchymal origin suggested by Simona Gurzu et al.^8^

Most GCTs are benign with slow growth, only 1–2% of all cases occur as malignant tumors which showed more nuclear pleomorphism, high nuclear to cytoplasmic ratio, and increased mitotic activity on histologic examination. In addition, malignant tumors tend to exceed 4 cm in size with invasion of adjacent structures or metastases.^1^ The coexistence of GCT and other malignant neoplasms in the same organ has been reported in multiple organ including tongue and larynx.^9^ Sometimes, biopsy or subtotal resection was too superficial to distinguish pseudoepitheliomatous hyperplasia (PEH) which appears on the mucosal layer in half of all cases of laryngeal GCTs from squamous cell carcinoma.^10^ In view of these problems, the treatment aimed at complete excision using laser or cold instruments and minimal functional damage. After complete resection, most cases can be cured, but recurrence still presents in 2–21% of all cases, usually at the primary site.^5^ Therefore, the patients need to be under follow-up with laryngoscopy to confirm complete recovery.

## Conclusion

In conclusion, laryngeal GCT is a rare and benign tumor. However, the possibility of coexistence with malignant neoplasm and voice disturbance were of concern. Complete resection with laser or cold knife for pathologic examination should be performed. To our best knowledge, this is the first case of glottic GCT in patients receiving levothyroxine supplement after total thyroidectomy. In this patient, although we cannot provide evidence that tumor growth is mediated by levothyroxine stimulation; more cases should be collected for analysis to understand the etiology of this rare tumor.

## Conflicts of interest

The authors declare no conflicts of interest.
